# History at the heart of medicine

**DOI:** 10.12688/wellcomeopenres.21229.1

**Published:** 2024-05-14

**Authors:** Richard T. Bellis, Fred Cooper, Rina Knoeff, Coreen McGuire, Manon Parry, Karin Tybjerg, Ruben E. Verwaal, Angela Woods

**Affiliations:** 1 University of St Andrews, St Andrews, Scotland, UK; 2 University of Bristol, Bristol, England, UK; 3 Rijksuniversiteit Groningen, Groningen, The Netherlands; 4 Durham University, Durham, England, UK; 5 Universiteit van Amsterdam, Amsterdam, The Netherlands; 6 Kobenhavns Universitet, Copenhagen, Denmark; 7 Erasmus Universiteit Rotterdam, Rotterdam, The Netherlands

**Keywords:** History, Collective memory, Collections, Imagination, Critical Medical Humanities, Embodiment, Critique, Body

## Abstract

With a focus on the challenges of today and tomorrow in the critical medical humanities the role of history is often overlooked. Yet history and medicine are closely intertwined. Right now, with the surfacing of knotty problems such as changing demographics, chronic pain, loneliness and Long Covid – and the consequent necessity to change directions and policies – history seems more urgent than ever. However, historians of medicine have sometimes been reticent to play a role in medicine and policymaking. The recent and welcome development of the critical medical humanities has intervened in medicine in important ways, but often without clear engagement with the history of medicine. In this letter, we make a renewed case for coherence and collaboration between history of medicine, medicine, and medical humanities, emphasising the continuity and links between all three. The skills and focus of the historian of medicine bring crucial historical context to the table, enabling better understanding of medical collecting, new imaginative futures, profound critiques of key medical concepts, and understandings of the body through time. By emphasising what historians can do for medicine and medical humanities, we call for building historical work into how medicine, illness and health are understood now and in the future. We suggest three potential roles for historians: keepers of memories, conversation partners, and futurist thinkers.

## Plain Language Summary

‘History at the Heart of Medicine’ was originally conceived of at a workshop at the Groningen Centre for Health and Humanities in June 2023. The authors worked together to answer how the history of medicine and medical humanities could be more closely integrated with each other and with medicine. We identified that the two fields are typically perceived to have different levels of willingness to engage with policy and intervene directly in medical practices. Although medical history has tended towards greater discretion in these respects, we found that history is a vital part of both medicine and the medical humanities. We make a positive case for history not as prescriptive, but as generative of possible worlds and imagined futures.

## Disclaimer

The views expressed in this article are those of the author(s). Publication in Wellcome Open Research does not imply endorsement by Wellcome.

## Introduction

**Figure 1.**
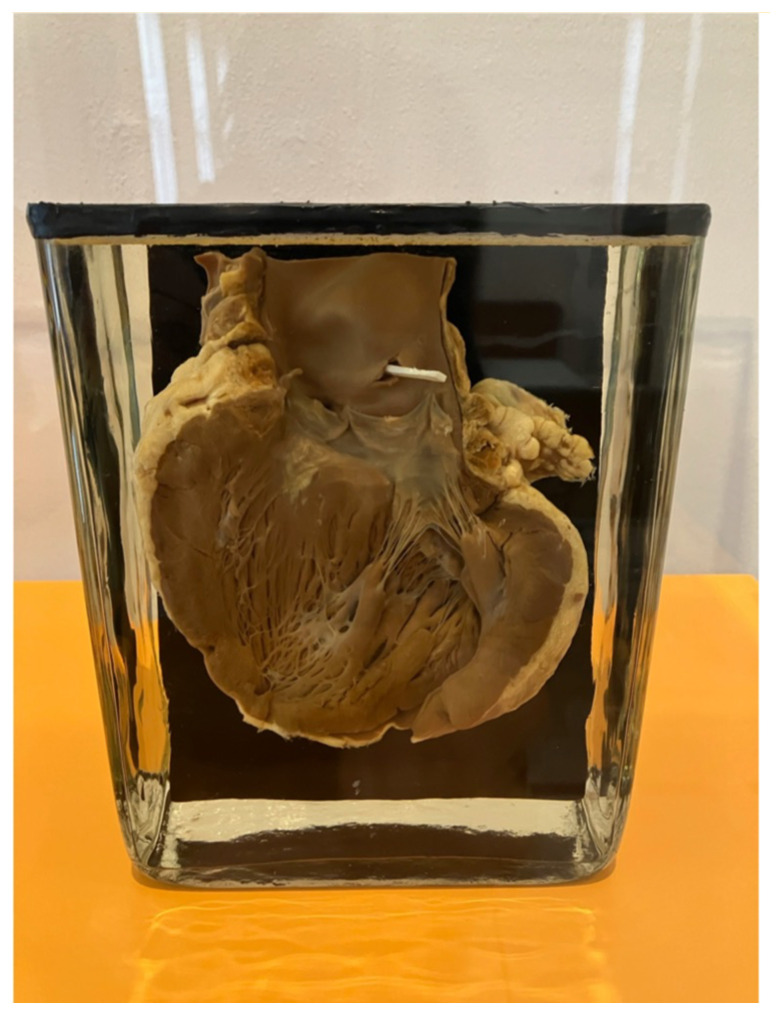
Heart of a 17-year-old woman who died from a ruptured aorta. Specimen from the Department of Forensic Medicine, University of Copenhagen, now in the Medical Museion, University of Copenhagen (reg no. 45: 2009 G). The white stick shows the rupture in the aorta. Photo Karin Tybjerg, reproduced with permission Karin Tybjerg and Medical Museion, University of Copenhagen.

This is the heart ( Figure 1) of a 17-year-old woman. Born in 1912, she was raised in an orphanage and worked as a domestic servant. She suffered from pneumonia and mumps in 1917, ‘Spanish Flu’ and diphtheria in 1919, whooping cough in 1920, and jaundice in 1924. In 1929 she went on a recreational stay and ran carrying a heavy suitcase. Soon afterwards she felt unwell, and suffered vomiting and pain in her chest. She died two days later from a rupture in the inner wall of her aorta.

This tell-tale heart holds a series of overlapping histories and temporalities, very literally inscribed on the body. Subject to a raft of conditions endemic to her time and place, the young woman was also caught in one of the century’s most virulent and fatal pandemics. The history of this heart – a history of epidemic and chronic illness, a life history, a history shared with millions of others yet uniquely experienced by each, an encultured, bodily, and metabolic history – opens up a set of questions which are of vital importance to the practice of medicine today.

What this heart tells us is that history and medicine are closely intertwined. Historians of medicine have generally been reticent about playing a role in medicine and policymaking, however, amid concerns over instrumentalization of the past, disciplinary rigour, ‘cherry-picking’, and inattentiveness to social and cultural differences and contexts ( Jordanova, 1995). There are exceptions to this of course; *History and Policy* and the *Journal of Applied History* are designed specifically to guide policy-makers, and many individual historians work closely with practising medics, scientists, and policy-makers. Yet the relevance of history in medicine, and even in the medical humanities, is often underappreciated.

This reticence and perceived irrelevance explain the often marginal place of historians of medicine in the critical medical humanities’ mission to highlight lived experiences of health, and to intervene in biomedical practices and debates. We write this piece to make a renewed case for coherence and collaboration, returning to the heart as an example which ties together multiple strands of our argument. We contend that medical history, medicine, and the medical humanities have far more in common than otherwise, and that now is the moment to unite.

## Collections

The heart above has its own specific history as a collected and curated object. Historical collections – objects, images, and archival sources – are material records of past decisions and practical considerations, each underscored by values and ideas; some active, like dissecting a heart, and some passive, like leaving it on a shelf, but all revealing. With respect to anatomical collections, engagements with human remains in the present can only be flawed and partial without understanding the histories of anatomical preservation and collection behind them ( Bellis, 2022; Tybjerg, 2022). The same goes for the origin of images and written accounts of the past. A closer interrogation of the past can also help navigate controversies – and silences – now. In the context of discussions about the use of human remains, this question is more urgent than ever.

Medical collections can variously promote comfortable narratives of progress but also, and often in the same space, ‘risky’ histories that challenge, provoke, and even discomfort ( Parry, 2020). What is found comfortable or uncomfortable reveals much about modern society and medicine. Historical work enables a broader range of narratives to be expounded, and for medical collections and archives to continue to be dynamic spaces for learning. The heart at the centre of this letter stands as an exemplar of what human remains can (sometimes) tell us: its clear value to our understanding of past pandemics and infectious diseases is unique. It is impossible to find a similar case ever again.

## Imagination

The account of the young woman captures our imagination, lending itself to narratives beyond the context of the medical information. It invites us to think otherwise about health and disease. How did the woman survive every single epidemic? How did they affect her? What was the secret of her resilience? Answers to these questions are an important antidote to what Mulgan identifies as a ‘crisis of the imaginary’, ‘the result of a deficit of social imagination’ ( Mulgan, 2020). Our capacity for futurism, in short, increasingly follows mainstream science fiction scripts of disaster, dystopia, or technological advancement; we find it far harder, however, to imagine sustainable and liveable futures. This imaginary crisis is a serious one, affecting how we respond to urgent challenges such as climate change, healthcare, and ageing.

As Tamson Pietsch and Frances Flanagan argue in relation to historicity and climate change, historical practice is an important tool to amplify imaginary thinking, showing – in the process of demonstrating how and why things are the way they are – how they might have, and still could, be different ( Pietsch & Flanagan, 2020). They disrupt what we consider to be evident, normal, or natural, a vital precondition for the new collective imaginaries, ideas, practices, orientations and values we need for the making of communities, institutions, and policies ( Knoeff, 2023). 

## Critique

The capacity for historical work to disrupt the normative dimensions of medicine has a second, interlocking function, best realised as part of what William Viney, Felicity Callard, and Angela Woods describe as the critical medical humanities, which have long prioritized the importance of lived experience alongside (and over) the limitations of biomedical knowledge ( Viney *et al*., 2015). The shared project we call for has to be resilient and capacious enough to make room for dissonance and critique, and this involves frank – but, we are sure, constructive – conversations over where respective disciplinary practices can learn, take stock, and transform.

Historical practice calls into question notions of ‘disease’ and ‘impairment’ as stable and self-explanatory entities, investigating categories of disability, neurodiversity, chronic illness, and disease not solely as biological realities, but as interactions between physical bodies, social environments, cultural attitudes, and economic processes ( McGuire, 2020). This requires the toolbox of the historian, which focuses on curating the past and includes skills such as historiography, palaeography, source critique and interpretation, archival work and object handling. Disability history, as a discipline firmly embedded in the politics, activism, and scholarship of the present, provides an excellent model for how historians of medicine might usefully situate and leverage their expertise, showing that concepts of health and disease are more contingent and less universal than often assumed, and contributing to more just societies and healthcare systems.

## Bodies

The heart specimen opens the question of how the body itself is historically situated and how history leaves traces in bodies. As the social epidemiologist Nancy Krieger has argued, ‘history is vital, because we live our history embodied’ ( Krieger, 2015). Indeed, history is important to medicine itself. Doctors take medical histories, and with respect to the young woman discussed above, the anamnesis was thought relevant to her death. This story of the heart resounds in today’s concerns about Long Covid. Epigenetics likewise reveals environmental effects over generations. Diseases of old age require studies of whole lifetimes through case notes, collections of pathological specimens, or aged biobank samples. The practice of medicine rests on a heavy silt, with disease and illness studied on the basis of past diagnostic categories; if our bodies, minds, and systems of treatment and knowledge are all products of history, the concerns of doctors and historians might be more closely related than each let on ( Keuck, 2018; Tybjerg, 2023).

## Conclusion

The heart from the introduction was dissected, preserved, labelled and stored because of the information it represented. The ruptured aorta was the end of one story, but not of the stories the heart can tell. It tells us about early twentieth-century life, illness, disability, and lived experience of disease; diagnosis, treatment, and death; the importance of community, familial, and state care; the role of stigma in the medical marketplace; and infection control within the material and media constraints of the period: why did she run two kilometres with a heavy suitcase? Answering such questions goes hand in hand with the material investigation of the heart’s matter. Through it we learn more about how to care for each other; the heart of the matter for both medical history and the medical humanities.

We envisage three roles for the historian in medical humanities: As *keepers of memories*, historians have the unique and necessary skills to read, interpret, translate, preserve, and protect writings, objects, visual representations etc. Left alone, these memories would be vulnerable and easily affected by changing moralities, sentiments, and policies as well as loss and decay. As *conversation partners*, historians bring to the table ways of critical thinking that question assumptions, structural problems, and historically grown truths underlying today’s concerns. And as *futurist thinkers*, historians contribute to imaginary thinking about future possibilities and scenarios. To be entirely clear, historians do not offer ready-made prescriptions for the future. Yet, they have a unique longue durée knowledge about social and cultural signals and patterns that can feed our imagination on what is possible (and perhaps also desirable). As a result, history does more than critically break down categories, it is also crucial in building anew.

Our hope is to lay common ground for a revised and re-energised conversation between archivists, curators, historians of medicine, scholars working under the loose umbrella of the medical humanities, as well as researchers and practitioners in medicine and public health. Many of the preconditions for this shift are already in place: in the meticulous and significant work that historians of medicine are already doing; in the flourishing and innovation of the medical humanities; and in the use of history for health and wellbeing. This is our call: to shift the terms of collective engagements with the past, building historical work into how medicine, illness and health are understood in the present, and imagined in the future.

## Ethics and consent

Ethical approval and consent were not required.

## Data Availability

No data are associated with this article.
