# Supplementary material for: History at the heart of medicine
Source: Wellcome Open Res. 2024 May 14;9:249. [Version 1] doi: 10.12688/wellcomeopenres.21229.1 (PMC11437318; doi:10.12688/wellcomeopenres.21229.1)
Supplement: Peer review history

# Reviewer report: Reviewer response for version 1

**DOI:** 10.21956/wellcomeopenres.23476.r98657

**Published:** 2024-10-09

**Authors:** Bernice L Hausman

**Affiliations:** 1 Penn State College of Medicine, Hershey, USA

The authors of the open letter on “History at the heart of medicine” begin with a claim that seems strange to me—that historians are absent from the medical humanities. From my vantage point in the US, history is very much a part of the medical humanities. Departments of the history of medicine are not uncommon in medical schools (Wisconsin, Ohio University, and Johns Hopkins come to mind). Additionally, many of us in the medical humanities teach with history intertwined with other disciplinary approaches. In this case, I think specifically of the work of Charles Rosenberg on the dramaturgy of epidemics, so useful in understanding our recent pandemic experiences. I also think of the work of Jacqueline Wolf on the history of breastfeeding, childbirth anesthesia, and Cesarean section—in which as a historian she attempts to make interventions into our present moment (which the authors claim historians are unlikely to do). Additionally, I think about anesthesia, which, as a specialty, is particularly interested in its own history and the history of anesthesia technologies. Many physicians are also “hobby historians,” which suggests the importance of the discipline to medical practitioners themselves. So I feel as if I am missing the implicit warrant of the letter—that historians are somehow absent from the medical humanities discussion.

 While it is true that medical historians often keep to their own professional societies and journals, and it is also true, as the authors suggest, that many historians are loathe to get into the policy fray, it does not seem altogether true to me that medical historians do not participate in the medical humanities. What I cannot know for sure is whether this situation differs in the UK and Europe, where the authors hold academic appointments.

 For me, the final statements about how the authors “envision three roles for the historian in medical humanities” (keepers of memories, conversation partners, and futurist thinkers) are puzzling. It feels a little weird for these authors to tell medical historians what their roles are. The abstract initially identifies that the medical humanities and its attitude toward history and historians might be the problem (“the role of history is often overlooked”). I think that critical medical humanities is the place to start, not a problem with the integration of history and historians.

 A different way to approach what the authors think is a historical gap in critical medical humanities scholarship would be to ask: What are the barriers to entry into critical medical humanities as it is currently conceived? Is ethics invited into the conversation as well? How might critical medical humanities, with its sense of itself as separate from medicine yet wanting to make an impact, be creating barriers to engagement by other disciplines that have been and continue to be engaged with medicine, medical education, and the experience of illness and health?

Does the article adequately reference differing views and opinions?

Partly

Are all factual statements correct, and are statements and arguments made adequately supported by citations?

Yes

Is the Open Letter written in accessible language?

Yes

Where applicable, are recommendations and next steps explained clearly for others to follow?

Yes

Is the rationale for the Open Letter provided in sufficient detail?

Yes

Reviewer Expertise:

Medical and Health Humanities, Cultural Studies, Feminist Theory

I confirm that I have read this submission and believe that I have an appropriate level of expertise to confirm that it is of an acceptable scientific standard.

# Reviewer report: Reviewer response for version 1

**DOI:** 10.21956/wellcomeopenres.23476.r94396

**Published:** 2024-09-27

**Authors:** monika pietrzak-franger

**Affiliations:** 1 British Literature and Culture, University of Vienna, Vienna, Austria

This is a well-argued case for a systematic inclusion of historical perspectives in the work of Medical Humanities. The authors highlight the triple function of historians -- "keepers of memories, conversation partners, and futurist thinkers" -- as central both to the field and to the policy making. I wholeheartedly agree that we cannot solve any current problems without consulting experts who have "a unique longue duree knowledge" about socio-cultural and political transformations and about the ways in which health and health care can and have been reimagined for the future.

Does the article adequately reference differing views and opinions?

Yes

Are all factual statements correct, and are statements and arguments made adequately supported by citations?

Yes

Is the Open Letter written in accessible language?

Yes

Where applicable, are recommendations and next steps explained clearly for others to follow?

Yes

Is the rationale for the Open Letter provided in sufficient detail?

Yes

Reviewer Expertise:

Medical Humanities

I confirm that I have read this submission and believe that I have an appropriate level of expertise to confirm that it is of an acceptable scientific standard.

# Reviewer report: Reviewer response for version 1

**DOI:** 10.21956/wellcomeopenres.23476.r97516

**Published:** 2024-09-17

**Authors:** Martin Bricknell

**Affiliations:** 1 King’s College London, London, UK

This is a nicely written short report which makes a compelling case to re-imagine the role of the historian in medical humanities. This has implications for departments of medical humanities, current medical historians, and students with an interest in medical history. As George Santana is quoted 'those who fail to learn from the past are doomed to repeat it'.

Does the article adequately reference differing views and opinions?

Yes

Are all factual statements correct, and are statements and arguments made adequately supported by citations?

Yes

Is the Open Letter written in accessible language?

Yes

Where applicable, are recommendations and next steps explained clearly for others to follow?

Yes

Is the rationale for the Open Letter provided in sufficient detail?

Yes

Reviewer Expertise:

Military medicine

I confirm that I have read this submission and believe that I have an appropriate level of expertise to confirm that it is of an acceptable scientific standard.
